# Roles of palmitoylation in structural long-term synaptic plasticity

**DOI:** 10.1186/s13041-020-00717-y

**Published:** 2021-01-11

**Authors:** Benjun Ji, Małgorzata Skup

**Affiliations:** Nencki Institute of Experimental Biology, 02-093 Warsaw, Poland

**Keywords:** Lipid posttranslational modification, Long term potentiation (LTP), Long term depression (LTD), Structural plasticity, AMPAR, Actin cytoskeleton, Rho GTPases

## Abstract

Long-term potentiation (LTP) and long-term depression (LTD) are important cellular mechanisms underlying learning and memory processes. *N*-Methyl-d-aspartate receptor (NMDAR)-dependent LTP and LTD play especially crucial roles in these functions, and their expression depends on changes in the number and single channel conductance of the major ionotropic glutamate receptor α-amino-3-hydroxy-5-methyl-4-isoxazolepropionic acid receptor (AMPAR) located on the postsynaptic membrane. Structural changes in dendritic spines comprise the morphological platform and support for molecular changes in the execution of synaptic plasticity and memory storage. At the molecular level, spine morphology is directly determined by actin cytoskeleton organization within the spine and indirectly stabilized and consolidated by scaffold proteins at the spine head. Palmitoylation, as a uniquely reversible lipid modification with the ability to regulate protein membrane localization and trafficking, plays significant roles in the structural and functional regulation of LTP and LTD. Altered structural plasticity of dendritic spines is also considered a hallmark of neurodevelopmental disorders, while genetic evidence strongly links abnormal brain function to impaired palmitoylation. Numerous studies have indicated that palmitoylation contributes to morphological spine modifications. In this review, we have gathered data showing that the regulatory proteins that modulate the actin network and scaffold proteins related to AMPAR-mediated neurotransmission also undergo palmitoylation and play roles in modifying spine architecture during structural plasticity.

## Introduction

### Structural long-term synaptic plasticity

Long-term potentiation (LTP) and long-term depression (LTD) are two forms of synaptic plasticity orchestrated by glutamatergic signaling that have been extensively studied and are considered to be cellular correlates of learning and memory processes [1–3]. Since the discovery of their important roles, a number of studies have been conducted using different experimental approaches both in vitro and in vivo in order to illuminate their mechanisms including induction, maintenance and links to learning and memory, as elegantly reviewed in [1–4]. Both ionotropic and metabotropic glutamate receptor activation were shown to be capable of initiating LTP and LTD via mechanistically similar but distinct signaling pathways [3, 5–7], of which *N*-methyl-d-aspartate receptor (NMDAR)-dependent LTP and LTD are regarded as the most prevalent forms of synaptic plasticity. Generally, long-term synaptic plasticity consists of both functional and structural components. Regarding the functional aspect, it is widely recognized that NMDAR-dependent LTP and LTD is primarily based on increasing or decreasing the number of α-amino-3-hydroxy-5-methyl-4-isoxazolepropionic acid receptors (AMPARs) on the postsynaptic membrane to modulate the strength of synaptic transmission via activity-dependent changes in AMPAR trafficking (reviewed in [1, 2, 4, 8–10]). AMPARs, assembled through different tetrameric combinations of four distinct subunits: GluA1, GluA2, GluA3 and GluA4 (previously known as GluR1-4), which are the major ionotropic glutamate receptors mediating fast excitatory neurotransmission, act as a gatekeeper for NMDAR-dependent synaptic plasticity by controlling Ca^2+^ permeability [2, 10–13]. This feature depends on whether the RNA-edited-GluA2 subunit is present within the tetramer (for more details see [2, 14–16]).

**Fig. 1:**
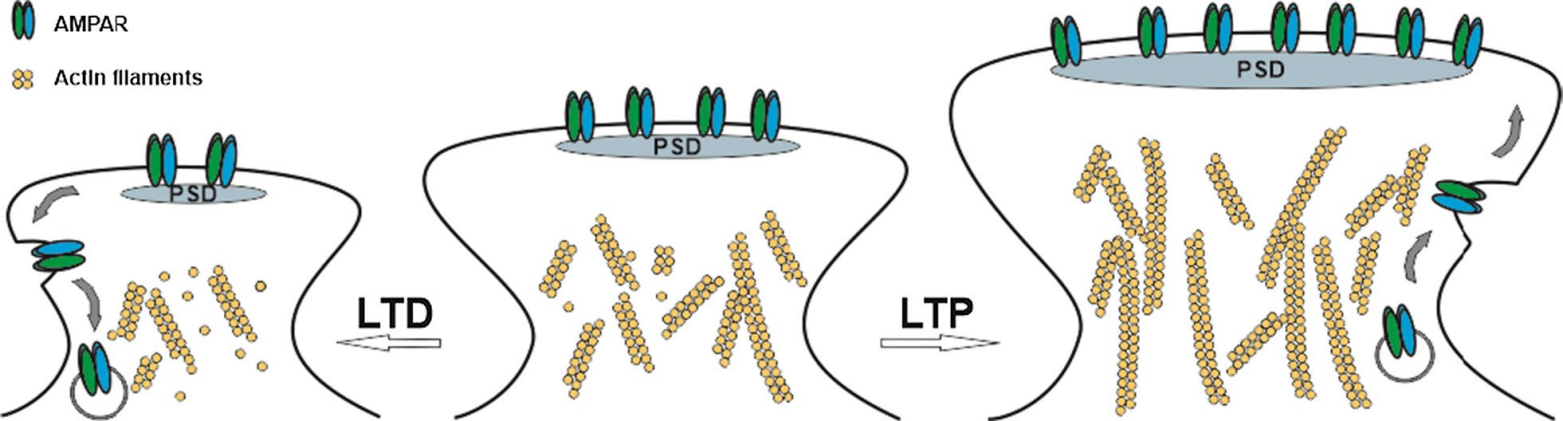
Spine enlargement in LTP and shrinkage in LTD. LTP triggers spine enlargement (the transient, fast increase to ~ 200 to 400% of spine head volume at 1–5 min, followed by ~ 50% increase persisting for over 1 h), which is supported by an increased actin cytoskeleton network within the spine [17–22]. Meanwhile, more AMPARs are recruited to the postsynaptic membrane through exocytosis and anchored on the postsynaptic density (PSD), which is also increased in the persistent phase of LTP. Conversely, LTD induces AMPAR endocytosis and spine shrinkage (a decrease by ~ 30% of spine head volume at 45 min after low-frequency stimulation) with depolymerization of the actin cytoskeleton network [18, 22, 23]

In addition to the functional consequences of quantitative changes in postsynaptic AMPA receptors in regulating synaptic neurotransmission, another particularly interesting aspect is the occurrence of structural plasticity (structural LTP and LTD); structural plasticity commonly accompanies functional LTP and LTD and is manifested by spine enlargement (LTP) [17–22] and shrinkage (LTD) [18, 22, 23] (Fig. 1). Morphological changes in dendritic spines have been reported to correlate with and serve as the structural basis for increased synaptic neurotransmission [17] or act as a synaptic tag for the consolidation of late phase synaptic plasticity [21, 24–26]. Therefore, structural plasticity underlies the morphological basis of memory and plays essential roles in learning and memory formation [27–29]. As small protrusions that form on dendrites, which are the primary postsynaptic loci to receive excitatory inputs in the brain, spines and their morphogenesis are crucial for signal transduction and neuronal connectivity influencing brain function [30]. During structural plasticity, spines are highly dynamic, demonstrating apparent morphological changes involving their shape, size and density, which are driven by protein signaling within the dendritic compartment [31]. At the molecular level, spine morphology is directly determined by actin cytoskeleton organization within the spine and indirectly coupled with the number and trafficking of AMPARs at the spine head (Fig. 1) [32–34]. Both aspects are regulated by a vast network of signaling proteins. The actin cytoskeleton serves as the crucial structural base beneath AMPARs and the postsynaptic density (PSD) molecules supporting the morphology of spines. Actin filaments are the major cytoskeletal components in dendritic spines that determine and shape the spine structure, undergoing polymerization and depolymerization to implement spine enlargement and shrinkage in response to stimuli during LTP and LTD, respectively [35–39].

Two-photon (2P) glutamate uncaging is widely used to study the mechanisms behind the dynamic morphological changes in dendritic spines during structural plasticity both in vitro [17, 40] and in vivo [41]. The development of this technique [42] allows for single dendritic spines to be selectively stimulated to undergo long-term morphological changes. Spine enlargement can be induced by high frequency glutamate uncaging in the absence of Mg^2+^ [17, 20, 43], while low-frequency uncaging results in spine shrinkage [41, 43, 44]. Simultaneously, in order to elucidate the spatiotemporal signaling dynamics during structural plasticity, fluorescence reporters have also been used. Fluorescence resonance energy transfer (FRET)-based sensors have been applied to visualize signaling with high resolution in space and time within a single dendritic spine, assayed by two-photon fluorescence lifetime imaging microscopy (2pFLIM) (reviewed in [45]). Therefore, the combination of 2P glutamate uncaging and FRET-FLIM provides a powerful tool to study the structural synaptic plasticity of dendritic spines, which has been adopted in many studies ([40, 46–48], see review [49]). Moreover, 2P glutamate uncaging-triggered spine morphological changes is a valuable model to study the roles of palmitoylation during spine structural plasticity [50].

### Palmitoylation and its role in the nervous system

Palmitoylation, myristoylation and prenylation [51–53] are common lipid modifications characterized by the type of lipid and site of attachment to protein residues. Palmitoylation acts as a sticky “tag” that can increase the hydrophobicity of proteins and facilitate their interactions with the hydrophobic lipid bilayer of plasma membrane and intracellular membranes of organelles or vesicles. This process is based on posttranslational attachment of palmitate (a 16-carbon saturated fatty acid) to proteins on cysteine (Cys) residues through the formation of a labile thioester bond, known as *S*-palmitoylation [54]. The most dramatic localization changes are found in cytosolic proteins, which, upon *S*-palmitoylation, acquire a hydrophobic anchor facilitating their membrane docking, compartmentalization and stability. Unlike myristoylation and prenylation, which are stable and permanent, palmitoylation is reversible due to the lability of its thioester bond [55, 56]. Therefore, palmitoylation is one of the most unique post-translational modifications due to its reversible nature that provides a novel mechanism for regulating protein membrane localization, trafficking and interactions.

Moreover, palmitoylation is particularly well-suited to regulate neurotransmitter receptors and other membrane proteins because it is not obligatorily coupled to protein translation, unlike myristoylation and prenylation, which usually occur co-translationally [57]. Thus, a given protein can still be modified in response to a given stimulus or in a particular subcellular compartment. Many key neuronal proteins are conserved between organisms that have very different degrees of behavioral complexity and cognitive abilities; evolution of post-translational palmitoylation sites and PDZ [postsynaptic density protein 95 (PSD-95)/Discs large/zona occludens-1]-binding protein–protein interactive motifs increased the regulatory potential of these proteins in vertebrates. It has been proposed that additional regulation of neurotransmitter receptors and their interactors, made possible by acquisition of evolved palmitoylation sites, acted as gain-of-function-mutations important for complex nervous system functions [57]. These are often accompanied by synaptic plasticity.

During neuronal development, protein palmitoylation facilitates axonal and dendritic growth and regulates differentiation by controlling both anterograde and retrograde protein transportation, which is critical for fiber development [58–62]. In neurons, palmitoylation dynamically regulates membrane localization and trafficking of proteins between the plasma membrane and intracellular compartments such as Golgi apparatus, endoplasmic reticulum (ER) and recycling endosomes during plastic changes that occur in the synapses of developing and mature neuronal networks (elegantly reviewed in [61, 63]). Taken together, protein palmitoylation plays crucial roles in many aspects of neuronal development and in control of neurotransmission and synaptic plasticity in the mature nervous system in physiological conditions; thus, not surprisingly, palmitoylation/depalmitoylation errors result in brain pathology related to neurodegeneration and neuropsychiatric diseases [61, 64]. The key marker for Huntington disease (HD), huntingtin (htt), is normally palmitoylated at Cys214 by huntingtin interacting protein 14 (HIP14), a palmitoyltransferase also known as ZDHHC17 [65, 66]. The pathogenesis of HD was investigated in a murine disease model where mutant htt with an expanded polyglutamine tract displayed decreased palmitoylation due to reduced interaction with HIP14, which consequently gives rise to inclusion body formation and toxicity in cortical neurons [65]. Amyloid precursor protein (APP) and β-Site APP cleaving enzyme 1 (BACE1) have also been identified as undergoing palmitoylation in neurons and are implicated in the pathogenesis of Alzheimer’s disease (AD) [67–69]. Likewise, disrupting palmitoylation of AMPAR has been associated with increased seizure susceptibility in palmitoylation-deficient knock-in mice with GluA1 palmitoylation site Cys811 replaced by Ser [70]. The manipulation of huntingtin, APP, BACE1 and AMPAR palmitoylation is a proposed therapeutic intervention for treatment of HD, AD or seizures.

Altered structural plasticity of dendritic spines is also a hallmark of neurodevelopmental disorders and accompanies psychiatric disorders including intellectual disability, epilepsy, autism spectrum disorder (ASD), schizophrenia and bipolar disorder, which display distinct spine pathologies such as aberrant dendritic spine density and morphology (see reviews [71–73]). Evidence has shown that palmitoylation is involved in some of these disorders exhibiting aberrant spine phenotypes. One common chromosomal abnormality that accompanies spine morphological deficits is 22q11.2 syndrome caused by a deletion of a small part of chromosome 22 where the ZDHHC8 gene, encoding a palmitoyltransferase ZDHHC8, is localized [74, 75]. In mice with 22q11.2 deletions, primary hippocampal neurons displayed decreased dendritic spines densities and glutamatergic synapses as well as impaired dendritic growth both in vitro and in vivo [76]. These deficits were prevented by reintroduction of the enzymatically active ZDHHC8 protein, indicating that lack of palmitoylation contributes to these deficits [76]. Further studies have revealed that ZDHHC8-dependent palmitoylation regulates structural plasticity of both axonal and dendritic spines, at least in part via Cdc42, which is a substrate of ZDHHC8 [77, 78]. The precise role of Cdc42 palmitoylation will be introduced later on in this review. In sum, these studies shed light on the importance of palmitoylation in structural plasticity and brain diseases.

Although the roles of palmitoylation in functional synaptic plasticity have been well documented [79–82], its roles in structural synaptic plasticity are not fully understood yet. As elucidated in many studies, structural plasticity is regulated by a vast network of signaling proteins. Within spines, signaling proteins need precise trafficking pathways and localization to implement their function in structural changes. Palmitoylation seems to play a key role in spatial and temporal control of the localization, compartmentalization and local abundance of these proteins. Therefore, in this review, we have gathered data to show the contribution and roles of palmitoylation of signaling proteins in the regulation of dendritic spine structural synaptic plasticity. We divided the proteins into three subclasses (Table 1): 1. main regulators of actin cytoskeleton modification, including Rho GTPases (Rac1 and Cdc42), LIMK1, δ-catenin and Ras GTPases (H-Ras and N-Ras); 2. AMPARs and their associated scaffold proteins [PSD-95, Ankyrin-G, ABP-L and AKAP79/150 (79 human/150 rodent)], which support and stabilize structural plasticity and link to the actin cytoskeleton; 3. candidate proteins including RhoB, Rab11, Rab8, Rabin8, Arc/Arg3.1, PICK1, SynDIG1 and β2-adrenergic receptor, whose palmitoylation may be linked to structural plasticity. We will focus on the first two subclasses of proteins whose palmitoylation has been more definitely shown to be involved in structural plasticity.

**Table 1:** Subclasses of palmitoylated proteins with different contribution to structural plasticity and enzymes catalyzing palmitoylation cycles

Protein	Palmitoylation sites	Palmitoyl acyl transferases (PATs)	Palmitoyl protein thioesterases (PPTs)	References
Main regulators of actin cytoskeleton modification				
Cdc42	Cys188, Cys189	ZDHHC3, ZDHHC8	–	[83–86]
Rac1	Cys178	ZDHHC3, ZDHHC8	–	[78, 87, 88]
LIMK1	Cys7, Cys8	–	–	[50]
δ-catenin	Cys960, Cys961	ZDHHC3, ZDHHC5, ZDHHC20	–	[89, 90]
N-Ras	Cys184	ZDHHC9	APT1, APT2, ABHD17,	[91–94]
H-Ras	Cys181, Cys184	ZDHHC9	APT1, APT2, PPT1, ABHD17B	[92, 94–99]
AMPAR and associated scaffold proteins which support and stabilize structural plasticity and indirectly link to actin cytoskeleton				
AMPAR	GluA1-Cys585, Cys811; GluA2-Cys610, Cys836; GluA3-Cys615, Cys841; GluA4-Cys611, Cys817	ZDHHC3	–	[86, 89, 90]
PSD95	Cys3, Cys5	ZDHHC2, ZDHHC3, ZDHHC7, ZDHHC8, ZDHHC15, ZDHHC17	ABHD17	[66, 76, 91, 96, 100–105]
Ankyrin-G	Cys70	ZDHHC5, ZDHHC8	–	[106–109]
ABP-L	Cys11	–	–	[83–85, 110, 111]
AKAP79/150	Cys36, Cys129	ZDHHC2	–	[50, 103–105]
Candidate proteins whose palmitoylation may contribute to structural plasticity and need to be further confirmed				
RhoB	Cys189, Cys192			[112]
Rab11	Cys223, Cys224	ZDHHC3, ZDHHC7	APT1, APT2	[113]
Rab8	–	ZDHHC2	–	[113]
Rabin8	–	ZDHHC3, ZDHHC7	APT1, APT2	[113]
Arc/Arg3.1	In _94_CLCRC_98_ motif	–	–	[114]
PICK1	Cys414	ZDHHC5, ZDHHC8	–	[115–117]
SynDIG1	Cys191, Cys192	–	–	[118, 119]
β2-AR	Cys265, Cys341	ZDHHC9, ZDHHC14, ZDHHC18	APT1	[120, 121]

## Palmitoylation-depalmitoylation dynamics

Palmitoylation-depalmitoylation cycling is catalyzed by palmitoyltransferases (PATs) and palmitoyl protein thioesterases (PPTs), respectively. Evidence indicates that the time course of the palmitoylation cycle can vary between minutes and hours. For instance, the half-life of palmitate on H-Ras and N-Ras approximately ranges from several minutes to 2.4 h [122–126]. In rat hippocampal neurons in culture, the half-life of glutamate receptor interacting protein 1b (GRIP1b) palmitate cycling was shown to be approximately 35 min [127]. This rapid turnover of palmitate on GRIP1b reversibly targets GRIP1b to dendritic endosomes and supports the role of GRIP1b in regulating AMPAR trafficking [127]. PSD-95 has a longer palmitate cycling half-life of approximately 2 h [128], indicating that palmitoylation of PSD-95 is more stable and suggesting a role in long-term static protein targeting. After chemical LTP (cLTP) in cultured rat hippocampal neurons, palmitoylation of δ-catenin, a component of the cadherin-catenin complex in dendritic spines, significantly but transiently increased, returning to basal levels by 3 h; in contrast, increased PSD-95 palmitoylation was maintained for up to 3 h in these conditions [89]. Taken together, these data strongly indicate that palmitoylation is a highly dynamic process, whose cycle reveals different kinetics dependent on the substrate protein.

PATs, a family of enzymes consisting of 23 members in mammals, are referred to as “ZDHHCs” according to current nomenclature (see recent review [80]). They contain a highly conserved DHHC motif (Asp-His-His-Cys) within a Cys-rich domain which serves as the enzyme that adds the palmitate to protein substrate. Biochemical and mutagenesis studies have shown that ZDHHC enzymes catalyze palmitoylation of substrates utilizing a ping-pong kinetic mechanism whereby the enzyme is transiently autopalmitoylated with palmitoyl-CoA (Pal-CoA), which can transfer the fatty acyl group to a free thiol present in cysteine residues of ZDHHC and then this palmitoyl-ZDHHC serves as an intermediate to transfer the palmitoyl group onto the substrate Cys residue [129, 130]. Most PATs have been localized intracellularly to the ER and Golgi apparatus [131], though there are some interesting exceptions, such as ZDHHC2, ZDHHC5 and ZDHHC8, which are found in dendrites and spines where they catalyze the palmitoylation of several signaling proteins including Cdc42, δ-catenin, PSD-95, Ankyrin-G-190, AKAP79/150 and PICK1 [77, 89, 105, 115, 132]. A specific amino acid region or signal sequence in ZDHHC PATs may determine the intracellular localizations of these proteins [133, 134]. Different C-terminal domains of ZDHHC2 and ZDHHC15 render different intracellular localizations of the two enzymes, and the lysine-based C-terminal sorting signals determine the restricted localization of ZDHHC4 and ZDHHC6 to ER membranes [133, 134].

Compared to the large PAT family, many fewer palmitoyl thioesterases have been identified. Acyl-protein thioesterase-1 (APT1) [95, 135], acyl-protein thioesterase-2 (APT2) [98, 136], palmitoyl-protein thioesterase-1 (PPT1) [97, 137] and palmitoyl-protein thioesterase-PPT2 [138] are the four well-known thioesterases (reviewed in [139]), which have distinct substrates (listed in Table 2). Notably, an elegant study demonstrated that microRNA-138-mediated knockdown of APT1 in neurons resulted in an accumulation of membrane-localized G protein Gα_13_ subunits that trigger RhoA signaling pathways to promote spine shrinkage, linking APT1 function to dendritic spine morphogenesis [140]. Hippocampal neurons from PPT1 knockout mice displayed structural and functional deficits, which include decreased dendritic tree complexity, lower dendritic spine density and fewer miniature excitatory synaptic currents [141]. Furthermore, PPT1 deficient mice exhibited a decreased ability to express LTP in the hippocampus than WT mice in response to tetanic stimulation [141]. In addition to APT and PPT enzymes, ABHD17 (α/β hydrolase domain-containing protein 17) was recently discovered as a depalmitoylating enzyme that removes palmitoyl chains from PSD-95, N-Ras and MAP6 ([91, 96, 142], see review [139]). These findings support the concept that the palmitoylation-depalmitoylation cycle plays important roles in the regulation of neuronal morphology and function and contributes to structural synaptic plasticity [141].

**Table 2:** Substrates of palmitoyl protein thioesterases

Palmitoyl thioesterases	Substrates
APT1	G protein α-subunit [95], H-Ras [95], N-Ras [93], Synaptosomal-associated protein 23 (SNAP-23) [143], endothelial nitric-oxide synthase (eNOS) [144], BK channel [145], β2-adrenergic receptor [120], sodium-calcium (Na-Ca) exchanger 1 (NCX1) [146]
APT2	GAP-43 [98, 147], H-Ras [98], N-Ras [93], ZDHHC6 [148], Scaffolding protein Scribble (Scrib) [149]
PPT1	H-Ras [97], Cysteine string protein α (CSPα) [150], Neurospecific peptides of G proteins α subunit, GAP-43, rhodopsin and myelin glycoprotein P_0_ [151, 152]
PPT2	Palmitoyl-CoA [138, 153]
ABHD17	PSD-95 [96], N-Ras [91], H-Ras [96], MAP6 [142]

## Palmitoylation of the main regulators of the actin cytoskeleton

In view of the fact that actin cytoskeleton sustains the formation and morphology of dendritic spines, the regulation of actin network within spines is particularly important. To remodel the actin architecture, many regulatory proteins and actin binding proteins (ABPs) play crucial roles through different signaling networks; among them, the most extensively studied are Rho GTPase signaling pathways, which play key roles in regulating spine morphology through modulation of the actin cytoskeleton [154]. Other Ras GTPases have also been identified as key modulators of actin network.

### Rho GTPases

The Rho GTPases, a subfamily of hydrolases of the Ras superfamily, are known for their essential roles in controlling actin cytoskeleton organization and dynamics in cells; therefore, Rho GTPases are able to regulate cell growth, migration, morphogenesis, survival and membrane trafficking [155–158]. Rho GTPases are activated by binding to guanosine triphosphate (GTP) and deactivated by binding to guanosine diphosphate (GDP), the process regulated by positive regulatory proteins: guanine nucleotide exchange factors (GEFs), and negative regulatory proteins: GTPase-activating proteins (GAPs) and guanine-nucleotide-dissociation inhibitors (GDIs) [159, 160]. GEFs turn-on signaling by catalyzing the exchange from G-protein bound GDP to GTP, facilitating dissociation of the tightly bound GDP and effectively leading to an increase in the number of GTP-bound molecules, GAPs terminate signaling by inducing GTP hydrolysis, while the main function of GDIs is to maintain their target Rho GTPases in soluble inactive complexes [160]. In neurons, Rho GTPases act as indispensable factors contributing to the organization of synaptic structure and morphology of dendritic spines and impacting synaptic neurotransmission, thus modulating synaptic plasticity [40, 161–164]. In the Rho family, Cdc42, Rac1 and RhoA are the three members most extensively studied and have been characterized with respect to their special roles at glutamatergic synapses. Cdc42 and Rac1 have been shown to promote spine formation and dendrite growth via the promotion of actin polymerization. Conversely, RhoA exhibits inhibitory functions in dendritic plasticity by destabilizing the actin cytoskeleton [162, 165]. Here, we will provide more details about Cdc42 and Rac1, which have been reported to undergo palmitoylation.

### Cdc42

Cdc42 promotes dendritic spine outgrowth, axon branching, morphogenesis and also contributes to the regulation of synaptic plasticity and learning and memory [78, 163, 166–169]. Cdc42 activation can be partially blocked by the inhibition of NMDAR or CaMKII, suggesting that Cdc42 proteins are partially activated by NMDAR/Ca^2+^/CaMKII signaling pathways [40]. Activation of Cdc42 is required for the delivery of AMPARs to the synaptic membrane to enhance synaptic potentiation during chemical LTP [170], through one possible signaling pathway that phosphorylates AMPAR GluA1 subunit at Serine 863 via a novel EphB2/Zizimin1/Cdc42/PAK3 (p21 activated kinase 3) signaling cascade [171]. Along with the effect of Cdc42 on AMPAR delivery, Cdc42 is responsible for regulation of spine morphology. In mice, Cdc42 conditional knockout in vivo under basal conditions led to a mild but statistically significant decrease in spine density in CA1 pyramidal neurons, suggesting a critical role of Cdc42 in the maintenance of spine morphology [166]. Similarly, the combined usage of 2P glutamate uncaging and FRET-FLIM at single dendritic spines of CA1 pyramidal neurons in cultured rat hippocampal slices caused a rapid and relatively sustained increase of Cdc42 activation, which was restricted to the stimulated spine heads, supporting spine enlargement [40]. Conversely, depletion of Cdc42 impairs structural plasticity as indicated by significantly reduced spine volumes [40, 166]. These results strongly demonstrate that Cdc42 plays an essential role in activity-dependent structural spine plasticity.

Cdc42 was found to exist as two isoforms: one is the canonical isoform, which is widely distributed, and the other is exclusively expressed in the brain [172]. Subsequent studies demonstrated that the canonical Cdc42 isoform is prenylated (Cdc42-prenyl), while the brain specific isoform is both prenylated and palmitoylated (Cdc42-palm) [83–85]. Cdc42-palm is preferentially concentrated in dendritic spines and plays a dominant role in regulating synaptogenesis, since knockdown of Cdc42-palm but not Cdc42-prenyl leads to significantly reduced spine inductions in cultured rat and mouse hippocampal primary neurons [83, 84]. It has been further demonstrated that the membrane localization of Cdc42-palm in dendritic spines is primarily facilitated by palmitoylation at Cys188, while palmitoylation at Cys189 plays a unique role in Cdc42-mediated spinogenesis [84, 85]. Upon synaptic activation, glutamate treatment in neurons caused a rapid depalmitoylation and dislocation of Cdc42 from dendritic spines [83]. In line with the above result, stimulation with AMPA also significantly induced Cdc42 deactivation and reduced membrane-located Cdc42 in rat primary cortical neurons in culture [173]. This phenomenon could be due to the finding that palmitoylation inhibits Cdc42 interaction with RhoGDI [85]. Namely, depalmitoylation of Cdc42 reverses this inhibition and increases the interaction between Cdc42 and RhoGDI and, in consequence, reduces a fraction of GTP-bound Cdc42 and blocks Cdc42 targeting to the membrane. Taken together, these results suggest dynamic palmitoylation/depalmitoylation cycling of Cdc42 that can be rapidly regulated by synaptic activity, most likely through an AMPAR activity feedback loop. On the one hand, Cdc42 activation on dendritic spine membranes could recruit AMPARs to synapses via specific signaling pathways to enhance neurotransmission [170]; on the other hand, AMPAR stimulation in turn dislocates Cdc42 from membrane compartments and inactivates Cdc42 [173]. This feedback loop involving Cdc42 may play crucial roles in the regulation of both structural and functional synaptic plasticity.

ZDHHC8 and ZDHHC3 are two PAT candidates for Cdc42-palm [78, 85]. Importantly, the ZDHHC8-dependent palmitoylation state of Cdc42 is involved in postsynaptic structural plasticity in neuronal diseases. Several studies demonstrated that 22q11.2 deletion syndrome leads to spine density deficits and impaired dendritic growth, which can be modulated by ZDHHC8 activity [76, 77, 174, 175]. In hippocampal organotypic slices from wild type mice, induction of ZDHHC8 overexpression causes a significant increase in spine density and spine stabilization, while knockdown of ZDHHC8 leads to the opposite effects. Consistently, overexpression of ZDHHC8 successfully restores spine density and stabilization in mice with 22q11.2 deletion syndrome to WT levels [77]. A particularly interesting observation of the study was that expression of the palmitoylated form of Cdc42 restored long-term spine stabilization in hippocampal slices from mice with 22q11.2 deletion syndrome, similar to another study on mice with that syndrome showing that palmitoylation of Cdc42 by ZDHHC8 was capable of promoting axon growth and branching [77, 78]. In sum, Cdc42 and its palmitoylation state act as active participants to affect both structural and functional synaptic plasticity by modulating spine morphology and neurotransmission.

### Rac1

Rac1 has been shown to play important roles in both structural and functional aspects of learning, memory and forgetting in different experimental models [176–179]. During spinogenesis, studied in cultured rat hippocampal neurons, overexpression of Rac1 increased the size of dendritic spines and recruited AMPAR clusters to newly formed dendritic spines, enhancing excitatory synaptic transmission [180] and indicating that Rac1 contributes to modulation of both the morphology and function of spines [180, 181]. Rac1 activation appears to affect the induction of both LTP and LTD in hippocampal synaptic plasticity [177]. Loss of Rac1 prevents LTP induction by selectively reducing synaptic AMPAR function [182]. In addition, microtubule-associated protein 1B (MAP1B)/T-lymphoma invasion and metastasis-inducing protein 1 (Tiam1, a Rac1GEF)—dependent Rac1 activation is required for AMPAR endocytosis and spine shrinkage during LTD [183]. This evidence indicates that Rac1 is essential in the regulation of NMDAR-dependent structural synaptic plasticity but the mechanism of its modulatory effect on AMPAR trafficking at postsynaptic membranes is unclear.

One well-studied signaling pathway of Rac1 contributes importantly to synaptic plasticity. Namely, NMDAR activation leads to translocation of Rac1 to postsynaptic densities where CaMKII phosphorylates the Rac1GEF kalirin-7 or Tiam1, which activates Rac1 locally [184–187]. Activated Rac1 transfers its activation to the Rac1/PAK/LIMK1 pathway to inhibit cofilin-mediated actin depolymerization and thus promotes actin polymerization [188–190]. In addition, during structural plasticity induced by glutamate uncaging at single spine of CA1 pyramidal neurons in organotypic hippocampal slices, FRET- FLIM imaging showed that protein kinase C isoform α (PKCα) knockout mice exhibits significantly attenuated Rac1 activation and deficits in structural plasticity compared with WT mice, indicating that Rac1 can also act as a downstream effector of PKCα to remodel actin cytoskeleton [191]. So how does Rac1 get to postsynaptic membranes to trigger these signaling events? Palmitoylation seems to be important in its targeting. Rac1 is palmitoylated at Cys178 in the C-terminus region, as shown in COS7 cells, murine embryonic fibroblasts and Jurkat T cells [88]. Palmitoylation of Rac1 translocates and stabilizes Rac1 at actin cytoskeleton-linked ordered membrane rafts (lipid rafts), which is required for cell spreading and migration [88]. In contrast, inhibiting Rac1 palmitoylation by mutation or 2-bromopalmitate reduces Rac1 localization in lipid rafts and Rac1 GTP loading, which combines with reduced activation of PAK at the plasma membrane [88, 192]. It is worth noting that PAK, one of the best characterized downstream effectors of Rac1, is crucial for cytoskeleton dynamics and cell morphology [193–195]. Therefore, palmitoylation seems to evoke Rac1 targeting to the plasma membrane and interaction with its effector to trigger downstream signaling pathways and facilitate actin remodeling in several model systems [88, 192]. In embryonic cortical neurons isolated from *Zdhhc8*-deficient mice, palmitoylation of Rac1 is reduced by 38% compared with that of WT mice, suggesting that ZDHHC8 is catalyzing palmitoylation of Rac1 in neurons [78]. So far, the exact role of Rac1 palmitoylation in neural synaptic plasticity is unclear.

Based on accumulating evidence, given that the palmitoylation of Rac1 occurs in synapses, we speculate that palmitoylation triggers Rac1 translocation to the postsynaptic membrane adjacent to AMPAR and its effectors, then activates effectors such as PAK signaling to modulate cytoskeleton architecture and AMPAR-dependent neurotransmission. There are several lines of evidence to support this hypothesis. First, Rac1 is suggested to be locally activated at the synapses in rat hippocampal neurons [196]. Secondly, several regulators of Rac1 have also been shown to localize at the synapses to implement their function: Rac1GEF Kalirin7 and Tiam1 interact with glutamate receptors and PSD-95 and regulate the actin cytoskeleton for altering dendritic spine morphology by activating the Rac1 signaling cascade, which is essential for structural LTP [185, 186, 197–201]. Then, the Rho GTPase activating protein 12 (ARHGAP12), the Rac1 GAP protein, at excitatory synapses is capable of reducing spine size and density through inhibition of Rac1 activity and promotion of AMPAR internalization by interacting with CIP4 [202]. Thirdly, the effector of Rac1, a phosphorylated PAK, is also locally activated and accumulated at synapses and colocalizes with PSD-95 in cultured rat hippocampal neurons [196], and synaptic AMPAR is associated with activation of the Rac1/PAK/ LIMK1 pathway that is necessary for actin-mediated spine enlargement during LTP [203].

These results suggest that Rac1 needs to be translocated towards the membrane to interact with its activators and effectors, which may be facilitated by palmitoylation. While investigating PATs for Rac1, two studies were found that showed Rac1 may be palmitoylated by ZDHHC3 and ZDHHC8 [78, 87]. However, involvement of palmitoylation in Rac1 translocation connected with spinal growth and identification of the PATs involved requires further experimental proof.

### LIMK1

Palmitoylation regulates not only localization of small Rho GTPases but also their downstream effectors. LIMK1 serves as a key downstream effector of the Rac1/PAK and Cdc42/PAK signaling pathways to regulate actin polymerization. A recent study showed that LIMK1 also undergoes palmitoylation, which is critical for the regulation of actin dynamics [50]. LIMK1 palmitoylation at Cys7 and Cys8 within a specific N-terminal motif targets and anchors LIMK1 to dendritic spine heads and enhances spine maturation and architecture by controlling the spine actin turnover; these effects can be abolished by CCSS mutation (Cys7/Cys8 mutated to non-palmitoylatable serine) and 2-bromopalmitate treatment [50]. Importantly, palmitoylated LIMK1 is required for activity-dependent spine enlargement in LTP induced by uncaging of glutamate, as spine enlargement deficits caused by LIMK1 knockdown could be rescued by WT LIMK1 but not LIMK1 with mutated palmitoylation sites [50].

Taken together, the data clearly demonstrates a sophisticated role for palmitoylation in the Rho GTPase signaling cascade mediating actin cytoskeletal regulation in dendritic spines. Palmitoylation of Cdc42, Rac1 and LIMK1 ensures a spatially precise, localized process for carrying out spine-specific actin regulation.

### δ-catenin

It has been demonstrated that δ-catenin can serve as an upstream regulator of Rho GTPases, exerting an influence on the actin cytoskeleton network [204]. δ-catenin also acts as a component of the cadherin-catenin complex, which plays important roles in the remodeling of dendritic spine morphology and synaptic structure [205–207]. Despite δ-catenin exhibiting different roles during various developmental stages, the loss of δ-catenin led to a reduction in spine head width and length in both developing and mature neurons [205–207], indicating δ-catenin is a crucial contributor to spine architecture. On the spine head, δ-catenin colocalizes and stabilizes cadherin at spine head plasma membranes to support cadherin function in activity-induced spine remodeling and anchoring AMPAR to postsynaptic membranes via cadherin-δ-catenin-ABP/GRIP complexes [208–213]. Inside the spines, δ-catenin appears to link to and regulate the actin cytoskeleton through an interaction with cortactin and modulation of downstream Rho GTPase signaling [204, 214, 215]. δ-catenin can be palmitoylated at Cys960 and Cys961 by both ZDHHC5 and ZDHHC20; however, activity-dependent palmitoylation of δ-catenin was shown to only require ZDHHC5 [89]. Neuronal activity leads to ZDHHC5 trafficking from the postsynaptic membrane to recycling endosomes in dendritic shafts to increase palmitoylation of δ-catenin after disrupting ZDHHC5’s interaction with Fyn and PSD-95, which stabilize ZDHHC5 in spine heads [216]. Subsequently, palmitoylated δ-catenin is trafficked back into spines to associate with N-cadherin and increase surface insertion of AMPAR and then stabilizes N-cadherin and AMPAR at the spine head [89].

In line with an increased number of surface AMPAR, palmitoylation of δ-catenin is also involved in structural remodeling of spines. δ-catenin knockdown by shRNA in cultured rat hippocampal neurons resulted in smaller and longer dendritic protrusions, which could be rescued by shRNA resistant WT δ-catenin but not palmitoylation-deficient mutant δ-catenin or N-cadherin-binding-deficient mutant δ-catenin. cLTP treatment enhanced the width of protrusions in shRNA resistant WT δ-catenin expressing neurons but not in shRNA resistant palmitoylation-deficient mutant δ-catenin or N-cadherin-binding-deficient mutant δ-catenin expressing neurons. One explanation for these phenomena appears to be that lacking palmitoylation results in failed localization of δ-catenin to the protrusions to interact with N-cadherin and downstream Rho GTPase signaling. These findings suggest that both palmitoylation of δ-catenin and N-cadherin binding are necessary for activity-dependent changes in spine morphology [89].

Furthermore, increased δ-catenin palmitoylation and δ-catenin-N-cadherin interactions were also observed in the contextual fear-conditioning paradigm, indicating that δ-catenin-palmitoylation-dependent and δ-catenin-N-cadherin-interaction-dependent structural and functional changes are also required in learning and memory [89]. Another intriguing finding showed that palmitoylation of δ-catenin in dorsal root ganglion (DRG) sensory neurons in rat models of neuropathic pain is dependent on ZDHHC3 rather than ZDHHC5 and ZDHHC20, which palmitoylate δ-catenin in hippocampal neurons [89, 90]. This difference implies either novelty in ZDHHC distributions or distinct mechanisms and roles of palmitoylation of δ-catenin in brain and spinal cord.

### Ras GTPases: Ras

Like Rho GTPases, Ras GTPases are also guanosine-nucleotide-binding proteins whose “on" and "off" states are controlled by binding to GTP and GDP respectively, whose exchange process is facilitated by GEFs and GAPs [160, 217]. There are four Ras isoforms: N-Ras, H-Ras, and the two K-Ras splice variants K-Ras4A and K-Ras4B, encoded by three *RAS* genes in humans [218]. Serving as binary molecular switches, these Ras isoforms are capable of controlling intracellular signaling networks and regulating actin cytoskeletal integrity, cell proliferation and migration [219–222]. Aberrant Ras signaling is implicated in oncogenesis and neurodevelopmental disorders [223–228]. In dendritic spines, Ras signaling plays important roles in structural plasticity. LTP induced by glutamate uncaging in pyramidal neurons in the hippocampal slices triggers robust H-Ras activation in the stimulated spines, which requires Ca^2+^ influx through NMDARs and CaMKII associated activation [46]. The persistent spine enlargement after LTP induction is correlated with an extent to which H-Ras is activated in the stimulated spine. Inhibiting the activity of H-Ras or its downstream (ERK) effectors reduced the magnitude of persistent spine enlargement [46]. This evidence indicates that Ras and its downstream signaling molecules are required for sustained spine enlargement during synaptic plasticity. Furthermore, activated H-Ras can diffuse away from the enlarged spine to support the structural plasticity of adjacent spines [46]. However, in contradiction to the above data, another study demonstrates an opposite role of Ras in structural plasticity [229]. Using a combination of 2P glutamate uncaging and FRET-FLIM, Ras activation in spines sustained by downregulation of neurofibromin, the major Ras inactivator in dendritic spines, was shown to impair spine structural plasticity and cause spine loss in an activity-dependent manner [229]. As discussed by the authors, in response to “different scenarios” of synaptic plasticity, Ras may activate different downstream signaling pathways to implement distinct functions on spine structural plasticity [229].

To activate downstream signaling pathways, Ras needs to associate with membranes; this process requires post-translational modifications of Ras such as prenylation and palmitoylation [230]. Prenylation serves as the initial step in transporting Ras to the membrane, and occurs at the cystine residue in the C-terminal CAAX (Cys-aliphatic-aliphatic-any amino acid) motif of Ras. Palmitoylation, as a second signal, occurs at Cys residue proximal to the C-terminal cysteinyl site of prenylation to stabilize membrane association of Ras [94, 231, 232] (Table 2). Although prenylation is indispensable for biological functions of Ras, without palmitoylation mislocalization of Ras occurs and Ras is unable to target plasma membrane properly to activate downstream signaling pathways [230, 233].

Palmitoylation and depalmitoylation cycle of Ras has been studied in different cell types, regulating the shuttling of Ras between intracellular (ER, Golgi apparatus and recycling endosomes) and plasma membranes [233–236] (PATs and PPTs in Table 2). Palmitoylated H-Ras and N-Ras by ZDHHC9/GCP16 (Golgi complex associated protein of 16 KDa) complex are shifted from the Golgi apparatus to the plasma membrane through the classical secretory pathway in vitro [92, 237]. Abrogating palmitoylation of N-RAS^G12D^ oncogene [substitution of the glycine (G) at position 12 by an aspartic acid (D)] perturbs hematopoiesis and prevents myeloid transformation in a murine cancer model, suggesting that palmitoylation of Ras can be a potential therapeutic target [238]. Although Ras has been shown to play important roles during structural plasticity, how palmitoylation of Ras participates in the process is still not fully understood. A very recent study has shown the function of Ras palmitoylation in the modulation of dendrite morphology [239]. Namely, ZDHHC9-mediated Ras palmitoylation is required for dendrite outgrowth and maintenance [239]. Knock down of Golgi- localized ZDHHC9 in primary rat hippocampal cultures leads to shorter and less complex dendritic arbors which can be rescued through expression of shRNA-resistant WT ZDHHC9 but not mutant ZDHHC9 with inactive palmitoylation function [239]. Similar result is observed when the effect of Ras knock down is rescued by expression of shRNA-resistant WT N-Ras but not the palmitoylation-deficient N-Ras (N-Ras C181S) [239]. These data indicate that the palmitoylating activity of ZDHHC9 and palmitoylation of N-Ras are required to regulate dendrite outgrowth. Knockdown of ZDHHC9 decreases N-Ras palmitoylation concomitant with a decrease in phosphorylated ERK, while overexpression of ZDHHC9/GCP16 increases N-Ras palmitoylation and phosphorylated ERK [239]. These data indicate that ZDHHC9-mediated Ras palmitoylation regulates dendrite outgrowth and maintenance through downstream ERK signaling pathways.

## Palmitoylation of AMPAR and its associated scaffold proteins: support and stabilization of structural plasticity and links to the actin cytoskeleton

**Fig. 2:**
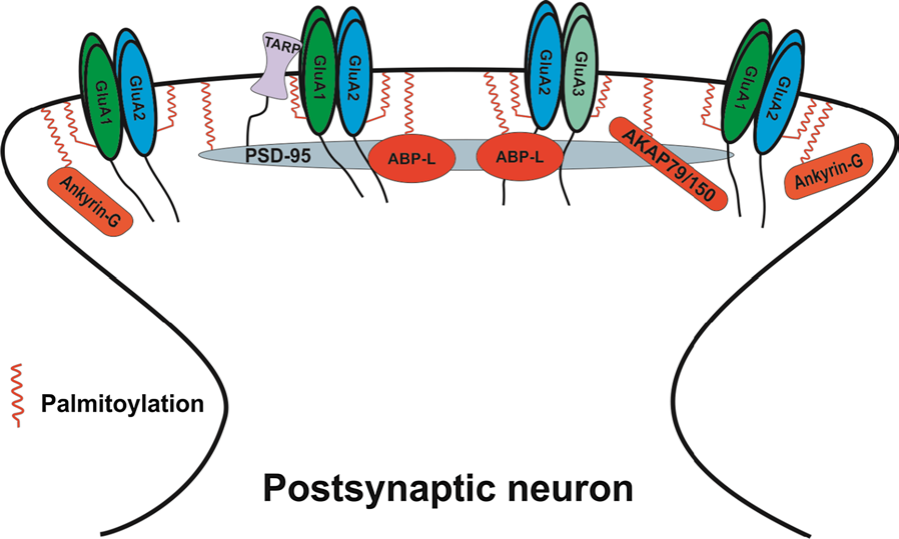
AMPAR and its associated scaffold proteins whose palmitoylation contributes to the modulation of spine morphology*.* The figure shows that several associated scaffold proteins of the AMPAR GluA1/2 and GluA2/3 heterotetramers (the major combinations of functional AMPARs in nervous system) undergo palmitoylation in the postsynaptic side of the excitatory synapses to regulate AMPAR membrane trafficking and postsynaptic architecture. Noteworthy, Ankyrin-G was shown to partially colocalize with GluA1 puncta perisynaptically in the spine head but the interaction between Ankyrin-G and GluA1 seems to be indirect and could be mediated by multiple proteins [244]. Palmitoylation of these proteins appears to play important roles in regulating their membrane localization and affects AMPAR trafficking on the postsynaptic membrane. It also contributes to spine structure modulation through various signaling pathways, which are described in this chapter. TARP: transmembrane AMPA receptor regulatory protein. One squiggle denotes one or more palmitoyl chains attached to the targeted protein. PSD-95 line icon denotes several PSD-95 molecules

The main regulators of the actin cytoskeleton, described above, directly initiate and drive remodeling of the actin network within the spines to affect spine architecture upon synaptic activity. In addition, on the spine head surface, the insertion and removal of AMPARs at the spine head not only alter neurotransmission but also contribute to the regulation of spine morphology [240]. Spine size appears to be linearly related to the number of AMPARs and the PSD area; this relationship is supported by the observation that large mushroom spines contain more AMPARs and have a larger PSD area compared to thin spines and filopodia [241–243]. Based on the accumulated evidence, AMPARs and their associated scaffold proteins (PSD-95, Ankyrin-G, ABP-L and AKAP79/150) (Fig. 2) play roles in the stabilization and consolidation of the persistent spine changes in a later phase of synaptic plasticity.

### AMPAR

In addition to their extensively studied role in synaptic neurotransmission [9, 10], AMPAR content of the postsynaptic membrane can also be linked to structural plasticity of dendritic spines [32, 245]. Many studies have indicated that, after the induction of LTP chemically or with two-photon uncaging of glutamate, a persistent increase in AMPAR-mediated synaptic neurotransmission was accompanied by a persistent increase in spine size [17, 20, 203, 246, 247], illustrating a tight correlation between AMPAR content in spines and spine morphology during synaptic plasticity. However, the relative timing of AMPARs recruitment associating with spine enlargement during LTP is still not fully clear. Some evidence demonstrates spine enlargement occurring earlier than accumulation of AMPARs on the spine surface in the initial phase of LTP [21, 247, 248]. In contrast, another study showed that AMPARs recruited to spines rapidly at the same time as the spine volume increased (within ∼10 s) after LTP induction [249]. In the initial phase, F-actin and its regulatory proteins including cofilin, Arp2/3 and Aip1 were shown to be massively transported to the spines to remodel actin cytoskeleton [21], while the ADF/cofilin-mediated actin dynamics are also capable of regulating AMPAR trafficking via a signaling pathway which is distinct from actin’s structural role in spine morphology [250].

Nonetheless, AMPAR GluA1 was shown to provide a link between spine enlargement and synaptic strength during LTP. Inhibition of Ca^2+^ permeable AMPARs (CP-AMPARs) by an antagonist prevented the persistent spine enlargement induced by chemical LTP [203]. Overexpression of a mutant GluA1 containing a C-terminal nonfunctional PDZ ligand inhibited LTP and GluA1 trafficking to the spines surface both in hippocampal slice cultures and in vivo; that manipulation also reduced the rapid spine enlargement induced by chemical LTP and prevented long-term stable spine enlargement, as compared to overexpression of WT GluA1 [251]. Interestingly, overexpression of GluA1 C-tail peptide also permitted chemical LTP-induced spine enlargement while a mutant GluA1 C-tail with a mutation in the PDZ ligand prevented spine enlargement [251]. These data suggest that synaptic insertion of GluA1 is required to stabilize and consolidate the spine enlargement through the structurally stabilizing effect of its C terminus, most likely on the actin cytoskeleton [251], since AMPAR can be linked to the actin cytoskeleton through several AMPAR-interacting proteins, including 4.1 N protein, actin depolymerizing factor (ADF)/cofilin, PICK1, ARP2/3 and Arc/Arg3.1 [245]. Also interesting is that the CP-AMPARs recruited by LTP are suggested to be upstream regulators activating the Rac/PAK/LIMK pathway that is responsible for actin-mediated spine enlargement, providing another functional link between AMPARs and spine morphology [203].

Palmitoylation of AMPARs has been demonstrated to functionally regulate its trafficking between the plasma membrane and intracellular compartments under basal conditions and during synaptic plasticity in a palmitoylation site- and subunit-dependent manner ([86, 252], recently reviewed in [80, 81]). Current data also implies that palmitoylation of AMPARs is involved in the regulation of structural plasticity. It is worth noting that 4.1 N protein, a neuron-enriched actin-associated protein, colocalizes with AMPAR and PSD-95 at the excitatory synapses on dendritic spines in primary hippocampal neuronal cultures [253, 254]. Through its association with GluA1, 4.1 N appears to facilitate GluA1 insertion and stabilize AMPARs in postsynaptic membranes [253–255]. Studies [86, 255] showed that surface expression of GluA1 was reduced after disruption of its interaction with 4.1 N or disruption of the actin filaments network. Further analysis demonstrated that palmitoylation on the C-terminal (Cys811) of GluA1 decreased the interaction of AMPARs with the 4.1 N protein and consequently led to AMPARs internalization. Conversely, via facilitation of phosphorylation at S816 and S818 residues, depalmitoylation of GluA1 Cys811 elevated GluA1 insertion into plasma membranes as a result of enhanced interaction between 4.1 N and GluA1 [255]. In mouse hippocampal slices, acute knockdown of 4.1 N significantly reduced late phase LTP expression, indicating that binding of 4.1 N to GluA1 is also required for LTP expression ([255], but see [256]).

Taken together, these results suggest that palmitoylation of AMPARs may negatively participate in the regulation of spine morphology during synaptic plasticity, weakening 4.1 N-mediated interaction with the actin cytoskeleton and cooperation with AMPAR phosphorylation. In line with this concept, a recent study provided more details about the relationship between AMPAR palmitoylation and spine volume [70]. Namely, under basal conditions, palmitoylation-deficient GluA1 C811S mutant mice did not show altered spine volumes compared with WT [70]. Glycine-induced chemical LTP led to spine enlargement both in palmitoylation-deficient GluA1 C811S mutant and WT groups; however, palmitoylation-deficient GluA1 C811S mutants displayed larger spine volumes compared with WT after LTP induction [70]. The latter may be caused by increased GluA1 insertion on the spine head with enhanced interaction between 4.1 N and GluA1 or through activation of the Rac/PAK/LIMK pathway. Currently, more details need to be obtained in order to answer to what extent and how palmitoylation of other AMPAR subunits regulates spine structural plasticity.

### PSD-95

PSD-95 is a member of the large membrane-associated guanylate kinase (MAGUK) family, which is enriched at the postsynaptic membranes of dendritic spines for the assembly of protein clusters and control of the localization and membrane trafficking of receptors, ion channels and associated signaling proteins. As a scaffold protein, PSD-95 protein serves as a binding “slot” located beneath the postsynaptic membrane that cooperates with stargazin to capture and immobilize AMPARs at synapses [257, 258]. Namely, the size of the PSD in the spine head directly determines and refines the numbers of AMPARs which can be accommodated and stabilized in the membranes. Overexpression of PSD-95 in rat hippocampal neurons in culture was shown to increase the number and size of dendritic spines and upregulate AMPAR levels at postsynaptic plasma membrane [259]. In early phase of LTP induced by 2P glutamate uncaging, PSD-95 keeps unaltered [21]. Until the late phase of LTP, which is protein synthesis-dependent, PSD size and PSD-95 were shown to increase proportionally to spine volume to consolidate LTP in persistently enlarged spines [21, 260]. On the other hand, chemical induction of NMDAR-dependent LTD results in rapid destabilization and removal of PSD-95 out of spine heads, accompanied with a slight but transient decrease in the size of the spine heads [261]. These data suggest that, PSD-95 plays a role in the consolidation of the spine enlargement rather than driving initial increase of spine size. Moreover, PSD-95 is, in turn, linked to actin filaments through associations with Shank, GKAP, SynGAP and Rac-GEF Kalirin-7 at the PSDs [198, 262–265].

Palmitoylation of PSD-95 on its conserved N-terminal Cys 3 and 5 is essential for PSD-95 stabilization within the postsynaptic density and is required for the clustering of associated receptors such as AMPAR to regulate synaptic strength ([102, 128, 261, 266, 267]; well-reviewed in [80]; Table 2). Upon activity, transportation of PSD-95 to spines and PSD-95 turnover at excitatory synapses both need palmitoylation, affecting postsynaptic structure. In visual cortical neuron cultures, NMDAR-activated BDNF-TrkB signaling, which plays important roles in long-term spine structural plasticity [19, 48, 268, 269], drives more PSD-95 into synapses and leads to enlargement of the PSD-95 puncta in dendritic spines [270, 271]. However, blocking palmitoylation of PSD-95 with 2-bromopalmitate abolishes the effect of BDNF on PSD-95 transport, indicating that palmitoylation of PSD-95 is required for the process [267, 271, 272]. Palmitoylation facilitates the targeting of PSD-95 into intracellular membrane compartments and trafficking of PSD-95 with microtubule-based vesiculotubular structures to spines [267]. After arriving at spines, PSD-95 turnover at the synapse also requires palmitoylation. At excitatory synapses in neurons, the palmitoylation of PSD-95 is highly dynamic. Continuous cycling between palmitoylation and depalmitoylation of PSD-95, initiated by local ZDHHC2 activity, defines subsynaptic nanodomains in each dendritic spine to orderly assemble PSD and anchor surface AMPAR and remodel postsynaptic nanodomains and architecture [273]. These assembled PSD-95 nanodomains were shown to determine the size of the PSD and synapses since large synapses with additional ZDHHC2 inserted into the spine membrane are found to contain more nanodomains [273].

At synapses, through PSD-95 interacting proteins, palmitoylation of PSD-95 appears to indirectly affect actin cytoskeleton modification. CDKL5 has been shown to be required during dendritic spine morphogenesis and excitatory synapse stability both in vitro and in vivo [274–276]. Synaptic targeting of CDKL5 requires its binding to the N-terminal region (amino acids 1–19) of palmitoylated PSD-95 but not nonpalmitoylated PSD-95, as shown in cultured rat hippocampal neurons [275]. Furthermore, disruption of the interaction between CDKL5 and PSD-95 also leads to reductions in spine density, size and density of surface AMPARs, which is in line with the effect of CDKL5 downregulation [275]. Intriguingly, the inhibition of dendrite growth in cultured rat cortical neurons caused by CDKL5 knockdown was shown to be rescued by overexpression of Rho GTPase Rac1, which colocalizes with CDKL5 and acts downstream of CDKL5 [274].

**Fig. 3:**
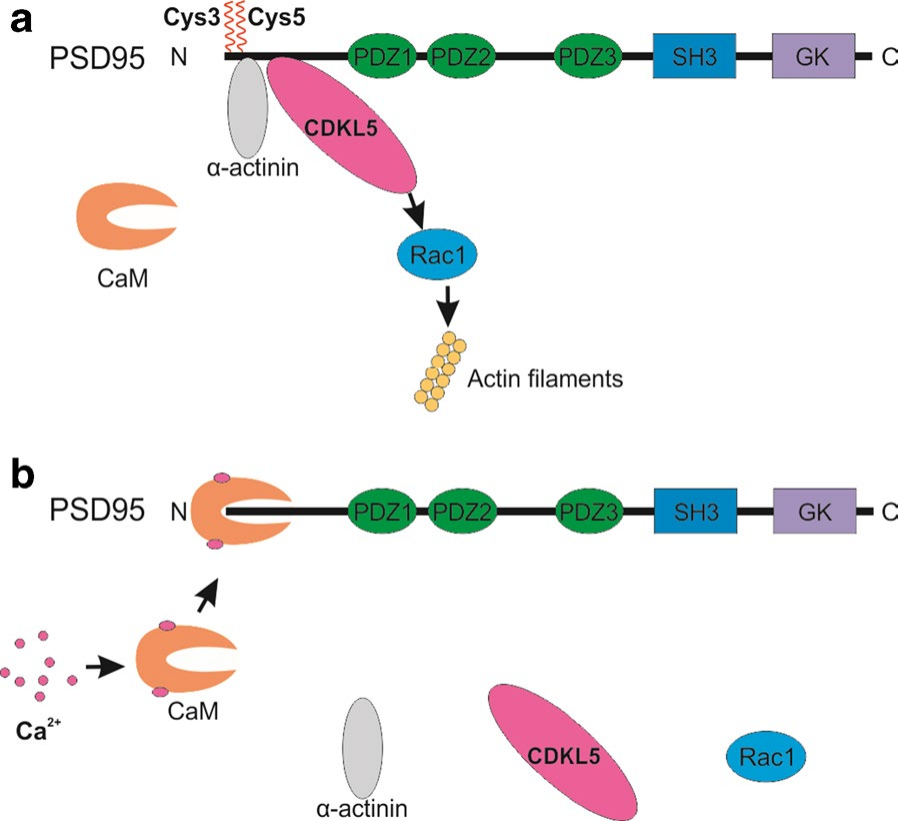
PSD-95 protein interactions are interrupted by Ca^2+^ influx which affects also PSD-95 relation with actin networks. A. PSD-95 is anchored on the postsynaptic membrane via palmitoylation at Cys3 and Cys5 and α-actinin anchoring. Through binding the N-terminus of palmitoylated PSD-95, CDKL5 is targeted to postsynaptic sites where it forms a complex with Rac1 to regulate the actin cytoskeleton via the Rac1 signaling pathway. B. Upon activity-induced Ca^2+^ influx through NMDAR, activated calmodulin (CaM), due to Ca^2+^ binding, caps the N-terminal of PSD-95, leading to blocked accessibility of palmitoylation sites and binding sites for CDKL5 and α-actinin. This process results in downregulation of PSD-95 palmitoylation and release of PSD-95 from the postsynaptic membrane. As a result, CDKL5 will also be released from the synapses and fail to form complexes with Rac1. Illustration based on [102, 274–278]. One squiggle denotes one palmitoyl chain attached to PSD-95

Taken together, these results provide a possible mechanism in which palmitoylated PSD-95 targets CDKL5 to the synapse where CDKL5 initiates Rac1 signaling-mediated actin remodeling and neuronal morphogenesis. F-actin binding protein α-actinin is another protein that binds to the N-terminus of PSD-95 to facilitate anchoring of PSD-95 at postsynaptic membranes but does not require and affect palmitoylation of PSD-95 [277]. In response to activity-induced Ca^2+^ influx through NMDARs at glutamatergic synapses, increased N-terminal capping of PSD-95 by calmodulin (CaM) blocks the accessibility of palmitoylation sites and binding sites for its partners, downregulating PSD-95 palmitoylation and dissociating CDKL5 and α-actinin from PSD-95 (Fig. 3) [277, 278]. This mechanism may explain a loss of synaptic surface PSD-95 and AMPAR during chemically induced LTD in cultured rat neurons [279]. A recent study showed that phosphorylation-dependent peptidyl-prolyl *cis*–*trans* isomerase (Pin1) binding to phosphorylated PSD-95 results in decreased palmitoylation of PSD-95, which subsequently led to a loss in the number of dendritic PSD-95 clusters, increased AMPAR mobility and a decreased number of functional excitatory synapses [280]. This data may shed light on the intertwined function of phosphorylation and palmitoylation of PSD-95 in the regulation of synaptic plasticity.

Recently, ABHD17 (α/β hydrolase domain-containing protein 17) was shown to be capable of depalmitoylating PSD-95 in neurons [91, 96]. Overexpression of ABHD17A/B/C in rat hippocampal neuronal cultures selectively downregulated PSD-95 palmitoylation and synaptic clustering of PSD-95 and AMPAR combined with decreased spine density and a decreased proportion of mushroom-like and filopodia-like spines [96]. These results also suggest that PSD-95 palmitoylation contributes to the regulation of spine architecture.

### Ankyrin-G

Ankyrin-G is also a multifunctional scaffold protein within the spines and is coupled to the actin cytoskeleton via spectrin. Encoded by the *ANK3* gene, Ankyrin-G contains diverse isoforms including small isoforms (100–120 kDa) and large isoforms, in which the larger isoforms (190, 270 and 480 kDa) are highly expressed in neurons [281–284]. Compared with the extensively studied roles of 270 and 480 kDa isoforms in axon initial segment (AIS), nodes of Ranvier (NoR) and inhibitory GABAergic synapses [109, 285–289], the Ankyrin-G 190 kDa isoform (Ankyrin-G-190) was shown to function in regulating the dendritic spine structure and glutamatergic neurotransmission ([244], Preprint [290]). At synapses, Ankyrin-G-190 forms subsynaptic nanodomains in the spine head surrounding PSDs and within the spine neck to stabilize dendrite and spine architecture both in vitro and in vivo ([244], Preprint [290]). Ankyrin-G-190 most likely serves as a perisynaptic scaffold associated with β-spectrin-actin cytoskeleton to support and stabilize the anchoring of perisynaptic AMPAR since Ankyrin-G-190 knockdown leads to a reduction of AMPAR GluA1 levels in spines and consequently reduces mEPSC amplitude [244]. During chemical LTP evoked in cultured rat primary cortical neurons, NMDAR activation drives Ankyrin-G-190 accumulation in spine subdomains and leads to spine enlargements, while knockdown of Ankyrin-G-190 fails to increase the spine size and density [244], indicating that Ankyrin-G-190 plays important roles in structural synaptic plasticity.

**Fig. 4:**
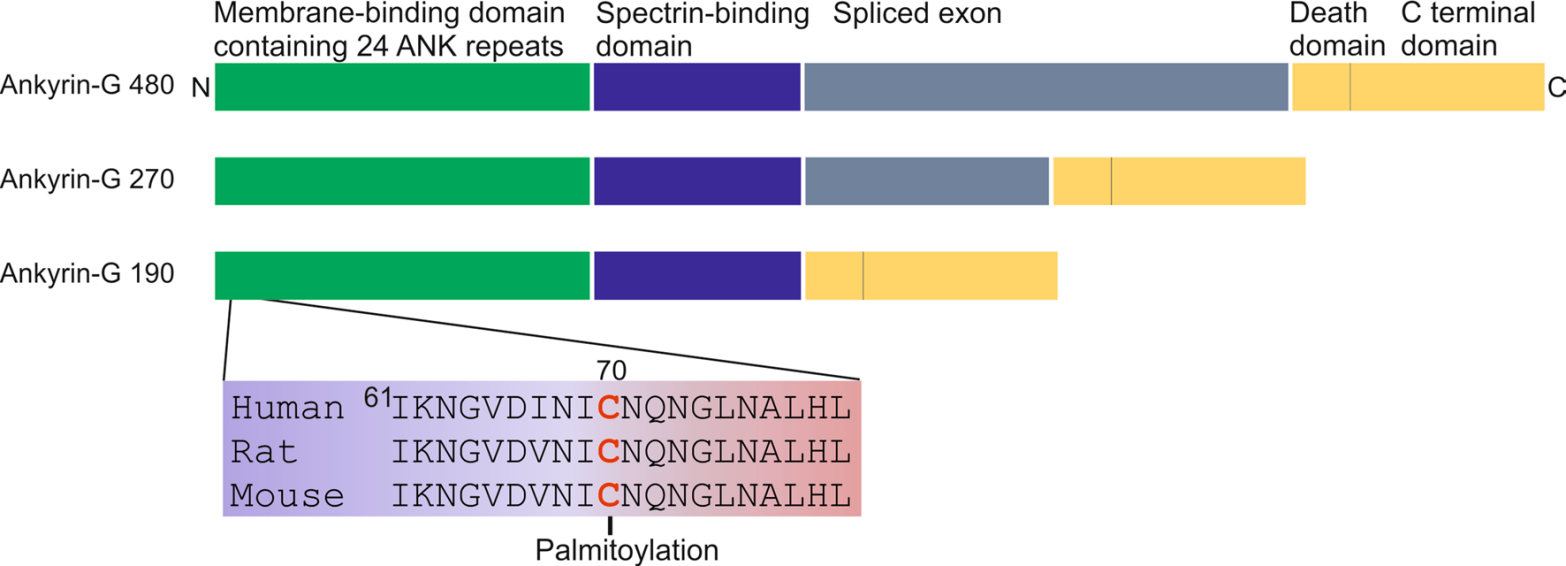
The Ankyrin-G isoforms highly expressed in neurons and localization of palmitoylation site in conserved membrane-binding domain. Ankyrin-G contains: membrane-binding domain, spectrin-binding domain and the death domain/C terminal regulatory domain. In addition to these three domains, Ankyrin-G 480-kDa and Ankyrin-G 270-kDa contain an extra spliced exon, a longer one and a shorter one, respectively. The palmitoylation site is a single conserved cysteine located in a loop of the first ankyrin repeat of its membrane binding domain [106, 281]

Ankyrin-G undergoes ZDHHC5/8-dependent palmitoylation at a conserved Cys70 (Fig. 4) in a loop of the first ankyrin repeat of its membrane binding domain in heterologous cells; that palmitoylation is required for association of Ankyrin-G with plasma membrane by forming a stable defined binding interface on the lipid membrane [106–109]. Palmitoylation of the Ankyrin-G 270 kDa isoform is essential for its membrane localization in AIS [106], while palmitoylation of the 480 kDa isoform has been shown to play an important role in stabilizing somatodendritic GABAergic synapses [109]. In unpolarized MDCK cells, a C70A mutation abolishes the association of Ankyrin-G-190 with lateral membrane, suggesting palmitoylation at Cys70 is required for cellular localization of Ankyrin-G-190 [106]. Of particular interest, a very recent study carried out in cultured rat primary cortical neurons showed that palmitoylation at Cys70 stabilizes Ankyrin-G-190 in spine heads and at dendritic plasma membrane nanodomains to maintain the dendrite and spine architecture (Preprint [290]). Mutated ankyrin-G-190 C70A displayed a diffuse distribution with decreased nanodomains in spine and dendrites (Preprint [290]). Differing from the Ankyrin-G-190 palmitoylation in MDCK and HEK293 cells [107], only ZDHHC8, but not ZDHHC5, catalyzes the palmitoylation of Ankyrin-G-190 in dendritic spines (Preprint [290]). Moreover, treatment with lithium, a commonly used mood stabilizer for treating bipolar disorder, selectively decreased the level of AnkG-190 palmitoylation and subsequently increased its mobility in dendritic spines by inhibiting ZDHHC8 action (Preprint [290]), indicating palmitoylation of AnkG-190 may also be involved in psychiatric disease.

Based on the current evidence, palmitoylation of Ankyrin-G-190 seems to play crucial roles in the maintenance of dendrite and spine morphology. Since Ankyrin-G-190 has been shown to contribute to spine enlargement and glutamatergic neurotransmission during LTP, it will be worthwhile to decipher more specific roles of Ankyrin-G-190 palmitoylation in these processes in future studies.

### ABP-L

Like PSD-95, AMPA receptor binding protein (ABP) is also a PDZ domain-containing scaffold protein, with two isoforms ABP-L (seven PDZ domain-containing) and ABP-S (six PDZ domain-containing) [111]. Due to its substantial similarity to GRIP1, ABP is also called GRIP2 [110, 111]. Both ABP and GRIP1 are shown to interact with AMPAR GluA2/3 subunits and regulate AMPAR membrane trafficking at synapses during synaptic plasticity [111, 291, 292]. Among them, the ABP-L and GRIP1b isoforms undergo palmitoylation which both occur at Cys11 in a very similar N terminus but have distinct functions [110, 127, 293, 294]. Compared with the role of GRIP1b palmitoylation in functional regulation of AMPAR trafficking, palmitoylated ABP-L (pABP-L) appears to regulate both synaptic structure and function. Different from the localization of palmitoylated GRIP1b in recycling endosomes in the dendritic shaft [127], pABP-L is targeted to spine head plasma membrane where it associates with surface-localized AMPAR GluA2 subunit [110, 294]. In contrast, non-palmitoylated ABP-L predominantly accumulates in the cell body and dendritic shafts, colocalizing with internal GluA2. More importantly, pABP-L was shown to be capable of modulating the spine structure to promote spine formation and maturation [294]. Overexpression of pABP-L in cultured rat hippocampal neurons induced more spines with larger spine head and shorter spine neck than non-palmitoylable pABP-L point mutant (C11A), indicating the ability of pABP-L to enhance spine maturation dependent on palmitoylation [294]. Besides the effects on postsynaptic spine morphology, pABP-L overexpression also increased the size of presynaptic terminals, indicated by larger synaptophysin puncta observed in pABP-L expressing neurons but not in non-palmitoylated ABP-L expressing neurons [294]. Along with both the pre- and postsynaptic structural changes, an increased AMPAR-mediated mEPSC amplitude and frequency and elevated surface AMPAR levels were also observed after pABP-L expression but not after the expression of the non-palmitoylated form of ABP-L [294]. These data strongly suggest that palmitoylation of ABP-L plays a crucial role in both structural and functional modifications of synapses. However, the exact role of pABP-L in activity-dependent structural and functional plasticity needs to be further investigated.

ABP-L has been well studied for its function in association with AMPAR to regulate AMPAR trafficking and is involved in long term plasticity, nevertheless, how pABP-L links to the actin cytoskeleton to remodel spine architecture remains uncertain. One possible pathway suggested by the authors [294] is through the interaction between pABP and δ-catenin [211, 294]. In this process, palmitoylation may facilitate the assembly of the complex containing pABP-L, δ-catenin, cadherin and PSD-95 by targeting pABP-L to spine heads [211]. In turn, δ-catenin interacts with cortactin to promote actin polymerization [214].

### AKAP79/150

AKAP79/150 (79 human/150 rodent) serves as a postsynaptic multidomain scaffold protein and regulates intracellular signaling events during long term synaptic plasticity by anchoring several protein kinases and phosphatases to postsynaptic structures to modulate AMPAR content on the postsynaptic membrane [295–303]. Palmitoylation of AKAP79/150 at N-terminal Cys36 and Cys129 facilitates the targeting of AKAP79/150 to dendritic recycling endosomes where palmitoylated AKAP79/150 assembles signaling complexes containing PKA, CaN, MAGUKs and AMPAR, trafficking them to the postsynaptic membrane to regulate synaptic neurotransmission during long term synaptic plasticity ([103–105, 304], see review [80]). During structural plasticity, AKAP79/150 palmitoylation controlled by ZDHHC2 is required for synaptic recruitment of AKAP79/150 and spine enlargement following LTP [103, 105]. In response to cLTP, overexpression of WT AKAP79/150 in cultured rat hippocampal neurons results in a spine enlargement with increased mean spine area and spine to shaft ratio, whereas palmitoylation-deficient AKAP79/150 mutant cannot; in line with that, AKAP79/150 knockdown prevented cLTP-induced spine enlargement that can be rescued by WT AKAP79/150 but not palmitoylation-deficient AKAP79/150 mutant [103, 105]. During LTD, removal of AKAP79/150 from dendritic spines appears to lead to spine shrinkage depending on the effects of CaMKII-regulated depalmitoylation of AKAP79/150 and disruption of the interaction between AKAP79/150 and F-actin [305]. However, the precise mechanism of AKAP79/150 in regulating structural plasticity needs to be elucidated.

## Candidate proteins whose palmitoylation may contribute to structural plasticity

In addition to the main regulators of actin cytoskeleton modification and AMPAR-associated scaffold proteins, there are also other proteins whose palmitoylation has been discovered either in functional synaptic plasticity or in heterologous cell systems. As these proteins themselves have been demonstrated to play a role in structural plasticity, we may therefore expect that their palmitoylation state may implement a function in structural plasticity in future studies. In this chapter, we will illustrate how these proteins contribute to structural plasticity and try to flesh out the links between their palmitoylation and structural plasticity.

### Other small GTPases: RhoB, Rab11, Rab8

Evidence indicates that RhoB also implements important roles in structural plasticity. Induction of LTP by high frequency stimulation in rat hippocampal slices leads to increased level of activated RhoB which occurs in an NMDAR dependent manner [306]. Consistently, deletion of RhoB in mice impairs LTP, significantly reducing early phase LTP but not affecting the later phase LTP [307]. Compared with WT mice, RhoB deficiency leads to a reduced level of phosphorylated LIMK and after LTP induction it abolishes the increase of phosphorylated cofilin [307]. Structural changes of dendrite spines are also observed: lack of RhoB leads to decreased spine number, increased proportion of stubby relative to thin spines, with a concomitant increase of length, head and neck widths of spines [307]. Taken together, these data suggest that RhoB is required for dendrite and spine morphology as the regulator of downstream effector LIMK and cofilin.

Recycling endosomes are thought to play crucial roles in regulation of functional synaptic plasticity, recycling AMPARs from endosomes to cell surface to regulate synaptic neurotransmission [308]. In addition, membrane from recycling endosomes provides additional building material for spine growth and remodeling during structural plasticity [309]. Small GTPase Rab11 is a well-known steady state marker and regulator of recycling endosomes [310]. Expression of Rab11 dominant-negative construct in rat hippocampal neurons or their treatment with Rab11 shRNA leads to a marked decrease in the total number of protrusions and dendritic spines compared with neurons from WT mice [311]. Recently, a novel signaling cascade Cdk5–LMTK1–TBC1D9B–Rab11A has been discovered to control dendrite spine formation and function in murine primary neurons and in vivo [312]. In addition, Rab11 has been found to be required in BDNF-induced dendritic branching [313]. This set of evidence suggests that Rab11-dependent dendritic recycling participates in spine modeling through multiple ways.

In spines, another member of Rab GTPases, Rab8, is also implicated in the regulation of AMPARs cycling and control of spine size [314, 315]. Rab8 is required for AMPARs delivery into the spine surface locally within the spine and expression of dominant negative mutants (GDP-bound form) of Rab8 (Rab8dn) inhibits LTP expression. [314, 315]. Moreover, expression of Rab8dn results in a reduction in spine size, suggesting that Rab8 contributes to the maintenance of spine morphology [314]. Rabin8, a Rab8 GEF, has also been shown to regulate neurite outgrowth of PC12 cells both through coordinating with downstream Rab8, Rab10, and Rab11 and through a GEF activity-independent mechanism [316]. As a downstream phosphorylation target of NDR1/2 (nuclear Dbf2-related kinase 1/2), phosphorylated Rabin8 contributes to spine morphogenesis by reducing filopodia and increasing mushroom spines both in vitro and in vivo [317].

RhoB, Rab11, Rab8 and Rabin8 were shown to undergo palmitoylation; RhoB is palmitoylated at Cys189 and Cys192 residues [112]. Rab11, Rab8 and Rabin8 were first examined to be palmitoylated in HEK293 cells in vitro, then Rab8 and Rab11 palmitoylation was confirmed in mouse embryonic brains in vivo [113]. Further analysis shows that palmitoylation of Rab11 is required for correct intracellular localization in NIH3T3 cells [113]. In addition to the limited knowledge about the roles of palmitoylation of the three proteins, how their palmitoylation affects synaptic plasticity is still not known. Based on the roles of the proteins themselves in spine morphological modulations, we may deduce that palmitoylation directs them to proper intracellular localization to trigger multiple downstream signaling pathways to exert a force on actin network within spines, that will require extensive studies to verify in the future.

### Arc/Arg3.1

Arc/Arg3.1 (Activity-regulated cytoskeletal-associated protein, also known as Arg3.1) belongs to the immediate-early gene (IEG) family, which can be induced rapidly by neuronal activity and transported to and locally translated in activated dendritic synapses [318–320]. During the persistent phase of LTP in rat dentate gyrus in vivo, Arc/Arg3.1 was shown to stabilize F-actin at synaptic sites by maintaining inactive phosphorylated cofilin, an actin-associated protein that disassembles actin filaments [321]. The stabilization of the actin network is regarded as a signal for spine enlargement during LTP, indicating that Arc/Arg3.1 may stabilize spine structural changes during LTP. Via palmitoylation at three cysteine residues clustered in a short N-terminal motif _94_CLCRC_98_, Arc/Arg3.1 is able to interact with membranes in neurons [114]. Particularly, the palmitoylation of Arc/Arg3.1 is required for myocyte enhancer factor 2 (MEF2, a transcription factor controlling gene expression)-dependent synaptic depression, confirmed by results showing that palmitoylation-deficient Arc/Arg3.1 fails to trigger synaptic depression induced by MEF2 [114]. In line with this, an earlier study showed that, in response to MEF2 activation, Arc/Arg3.1 knockout mice were unable to display functional synapse elimination or a decrease in dendritic spine density, compared with WT mice [322]. Taken together, palmitoylation of Arc/Arg3.1 may play important roles in both functional and structural changes during MEF2-dependent synaptic depression, since palmitoylation-deficient Arc/Arg3.1 displayed partially similar effects compared with knockout of Arc/Arg3.1. However, the precise roles of Arc/Arg3.1 palmitoylation on spine morphology remain to be explored.

### PICK1

PICK1 (protein interacting with C kinase-1) is a scaffold protein and plays crucial roles in the modulation of NMDAR-dependent LTD by regulating synaptic AMPAR trafficking. In addition to these observations, evidence also indicates that PICK1 contributes to the modulation of spine morphology during synaptic plasticity through the linking of AMPAR to the actin cytoskeleton network [323, 324]. It has also been shown that, in dissociated rat hippocampal pyramidal neurons in culture, overexpression of PICK1 reduces spine size while knockdown of PICK1 displays opposite effects [323, 324]; these results corroborate its functional roles in LTD. Further work uncovered a molecular mechanism, that PICK1 modulates spine morphology by inhibiting Arp2/3-mediated actin polymerization through binding F-actin and Arp2/3 complex, and the inhibition effects can be regulated by the activity of small GTPase Arf1 [323–325]. In addition, PICK1 was also found to interact with Cdc42 and form a triple complex with GluA2 in vivo [173]. AMPAR stimulation by AMPA treatments resulted in deactivation of Cdc42 in cultured rat primary cortical neurons that depended on PICK1 linking Cdc42 to AMPAR in the complex [173]. Since Arp2/3 complex can be activated by N-WASP, the downstream effector of Cdc42, these data suggest another PICK1 pathway, related to spine actin regulation.

ZDHHC8-dependent palmitoylation of PICK1 at Cys414 has been demonstrated to be required for the induction of cerebellar LTD in cultured mouse Purkinje neurons [115]. Inhibition of palmitoylation by 2-bromopalmitate, C414S mutation of PICK1 or ZDHHC8 knockdown by shRNA can all inhibit cerebellar LTD [115]. Palmitoylation was revealed to facilitate PICK1 anchoring on the postsynaptic membrane, confirmed by a recombinant construct mimicking constitutive PICK1 palmitoylation. Thus, palmitoylation may stabilize PICK1’s interactions with AMPAR and other binding proteins for AMPAR endocytosis and spine dynamics [115]. In spite of these developments, the precise roles of palmitoylation of PICK1 in different neuron types during structural plasticity still need to be explored.

### SynDIG1

SynDIG1 (synapse differentiation induced gene 1) is a novel type II transmembrane protein which has been identified in recent years as regulating synaptic development [119, 326]. SynDIG1 was shown to colocalize with AMPA receptors at excitatory synapses and interact with AMPAR through the C terminus of SynDIG1 in heterologous cells and neurons [119]. Although current evidence indicates that SynDIG1 probably contributes to excitatory synapse development through regulating AMPAR at synapses in a novel way [119, 326, 327], the exact mechanism of SynDIG1 regulating synaptic plasticity is still not fully clear.

Knockdown of SynDIG1 by shRNA in dissociated rat hippocampal neurons reduced the number and size of mature excitatory synapses, while SynDIG1 overexpression resulted in the opposite effects [119], suggesting SynDIG1 may have roles in regulating spine outgrowth. In vivo, SynDIG1-deficient synapses were found to be structurally immature in SynDIG1 homozygous mutant mice, indicated by a significant decrease in PSD length and the number of perforated synapses [326]. Upon LTP stimulation at single spines of hippocampal CA1 pyramidal neurons using 2P uncaging of glutamate, in WT mice, small spines displayed increased spine volume, while large spines did not change. However, in SynDIG1-deficient mice, although small spines tended to increase in spine volume, the more interesting observation was that large spines increased spine volume significantly in early-phase LTP but dropped down to initial volume late-phase [326]. Although this short-term and non-long-lasting enlargement of SynDIG1-deficient large spines may be explained by the evidence that SynDIG1 is required for spine maturation [326], the precise mechanism of how SynDIG1contributes to spine structural remodeling remains uncertain. Additionally, the question of why this hyperreactive enlargement occurs on SynDIG1-deficient large spines but not on small spines needs to be answered by further studies. SynDIG1 undergoes palmitoylation at C191 and C192 in the juxta-transmembrane region and the palmitoylation regulates the localization and stability of SynDIG1 at synapses [118]. Activity blockade by tetrodotoxin (TTX) enhances SynDIG1 palmitoylation and leads to increased SynDIG1 accumulation at synapses [118, 119]. Compared to WT SynDIG1s, which clustered at distal dendrites displaying enriched colocalization with endosomal marker EEA1 in dissociated rat hippocampal neurons, palmitoylation-deficient SynDIG1 mutants were restricted to the cell soma and proximal dendrites. Palmitoylation-deficient SynDIG1 mutants displayed enriched colocalization with ER and Golgi markers, suggesting palmitoylation may regulate SynDIG1 trafficking in the secretory pathway [326].

Thus far, the knowledge of the roles of SynDIG1 palmitoylation in synaptic plasticity is still lacking. LTP and LTD initiated through NMDAR activation triggers AMPAR membrane trafficking and morphological changes of spines. How the palmitoylation state of SynDIG1 participates in and regulates synaptic strength and spine structure during the induction and maintenance of LTP and LTD will be worthy to dissect, especially as data shows that LTP is abolished in 2-week-old SynDIG1-deficient mice [326].

### β2-adrenergic receptor

β2-adrenergic receptor (β2-AR) is a canonical G protein coupled receptor (GPCR), which functionally regulates AMPAR-mediated neurotransmission via β2-AR/PKA/GluA1S845 phosphorylation signaling pathway and contributes to LTP induction [328–332]. Whether or how β2-AR contributes to spine structural changes is largely unclear, but several studies provide evidence suggesting β2-AR may be related to the morphological changes of spines during synaptic plasticity and in diseases. One study showed that exposure to an enriched environment resulted in enlarged dendritic spines, enhanced hippocampal LTP and rescued the synaptic impairment by human Aβ oligomers in murine hippocampal slices, which partially required the activation of β2-AR/PKA signaling pathway [333]. As a result, an increased level of GluA1 pS845 and increased frequency of miniature EPSCs were observed [333]. In APP/PS1 (amyloid precursor protein/presenilin 1) transgenic mice, an AD animal model, β2-AR activation restored the density of spines and branches of dendrites and alleviated hippocampal memory deficits [334]. Another study demonstrated that deletion of β2-AR reversed the loss of dendritic spines and synapses and improved learning and memory through decreasing tau hyperphosphorylation, which is caused by Aβ induced β2-AR-PKA-JNK pathway in APP/PS1 mice [335]. Nonetheless, these results provide for the possibility that β2-AR signaling is involved in structural changes of spines.

Palmitoylation of β2-AR at Cys341 and Cys265 is suggested to facilitate the anchoring and stabilization of β2-AR at the plasma membrane, and subsequently affect the association with its interacting proteins ([120, 121, 336], see review [337]). The ability to couple β2-AR to the adenylyl cyclase signaling system is regulated by palmitoylation at Cys341 and PKA-dependent phosphorylation of β2-AR [336, 338, 339]. Golgi-resident ZDHHC9/14/18 and the plasma membrane-localized APT1 were shown to catalyze the palmitoylation and depalmitoylation of β2-AR at Cys265, respectively, suggesting novel trafficking of β2-AR between Golgi apparatus and plasma membrane regulated by the dynamic palmitoylation/depalmitoylation cycle [120]. Although current data on β2-AR palmitoylation has been gained mostly from studies in heterologous cells, it sheds light on the possible role of β2-AR palmitoylation in the β2-AR/PKA/AMPAR signaling pathway during synaptic plasticity by facilitating the anchoring of β2-AR on spine head surface adjacent to AMPAR to trigger downstream signaling which modulating synaptic strength and structure. While its exact roles in structural plasticity of dendritic spines needs to be determined by future studies.

## Conclusions and perspectives

**Fig. 5:**
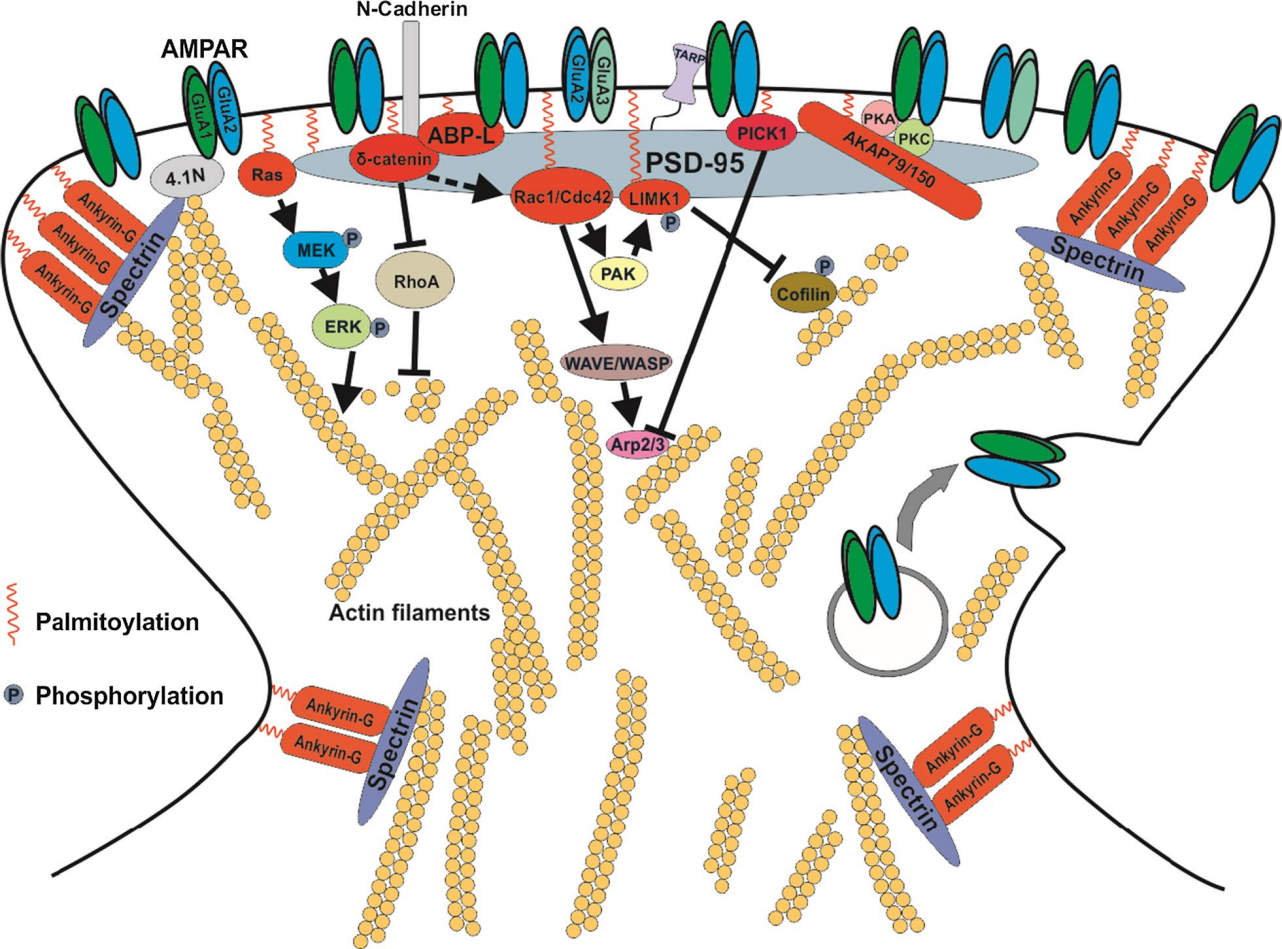
Roles of palmitoylation during structural LTP. During LTP, more AMPARs are recruited to the postsynaptic membrane via exocytosis and anchored on an enlarged PSD and on Ankyrin-G, which more densely populate the submembrane region than in the basal state. Ankyrin-G molecules are targeted to perisynaptic sites of the spine head and spine neck to stabilize the spine structure through palmitoylation and binding to spectrin (1:1), which is a cytoskeletal protein that lines the intracellular side of the plasma membrane interacting with actin cytoskeletal protein. AMPAR, 4.1 N, spectrin, actin and associated molecules form a membrane-associated periodic skeleton (MPS) structure observed in spines, but the extent to which it exists and how it develops in dendrites remain unclear [340, 341]. Palmitoylation targets Rac1, Cdc42, Ras and LIMK1 to spine membrane where Rac1 and Cdc42 activate PAK. Next, phosphorylated LIMK1 by activated PAK phosphorylates Cofilin and inhibits its depolymerizing activity. Palmitoylated Ras seems to participate in spine morphological modification through downstream MEK/ERK signaling pathway. In addition, through downstream WAVE and WASP, Rac1 and Cdc42 also regulate Arp2/3-mediated actin polymerization, which can be inhibited by PICK1. Palmitoylation also seems to facilitate assembly of the complex containing AMPAR, ABP-L, N-cadherin and δ-catenin on the postsynaptic membrane, linking AMPAR trafficking to the actin cytoskeleton network through Rho signaling. Regulation of functional and structural plasticity is a highly sophisticated process which needs precise timing of coordinated work of plenty of factors. Interplay between the processes described above and other, unknown factors, may lead to enhanced synaptic transmission and enlarged spines. The sketch was prepared based on the data collected in this review. One squiggle denotes one or more palmitoyl chains attached to the target protein; Spectrin line icon denotes several spectrin molecules

In this review, we reveal insights about protein palmitoylation’s role in the modification of spine morphology during structural synaptic plasticity (Fig. 5). Of note, almost all of the proteins we described above participate in both functional and structural aspects of synaptic plasticity, either regulating AMPAR trafficking or linking AMPAR and auxiliary proteins’ signaling to actin cytoskeleton remodeling. It supports the concept that functional and structural changes intertwine in most types of synaptic plasticity. Obviously, these changes are mediated by a set of proteins which work in coordination and most likely form a sophisticated network to regulate AMPAR trafficking and the actin cytoskeleton. However, how many proteins the complex contains and how they assemble spatially and temporally remains to be further explored. Known for its reversible and dynamic nature, palmitoylation plays important roles in controlling protein trafficking and targeting the transported proteins to the complex in postsynaptic spines.

Currently, in a majority of studies, research usually focused on single “bricks”—exploring the role of palmitoylation of a single protein mostly in vitro. Again, how palmitoylation spatially and temporally regulates the trafficking and localization of protein assemblies in vivo to build the higher order structures is an attractive question, awaiting a full answer. Palmitoylation is dynamically regulated by PATs and PPTs, but not all of the substrates of these enzymes have yet been identified. Additionally, discovery of enzymes that catalyze the palmitoylation/depalmitoylation cycling of already identified protein substrates and deciphering their kinetics will help us to build a more complete image of the dependence of synaptic plasticity on palmitoylation.

Structural plasticity is commonly accompanied by functional LTP and LTD, as shown in hippocampal neurons; though there are also studies showing that the expression of functional plasticity can be dissociated from structural changes, diverging in the downstream signaling events [21, 24–26]. Another exception can be found in a study showing that LTD in rat cerebellar Purkinje neurons did not display spine changes (number or size) when evaluated within one hour after induction [342]. Whether spine morphological changes may not accompany some forms of synaptic plasticity needs to be verified by additional studies. However, these exceptions could not negate the importance of structural plasticity of dendritic spines in the events related to learning, memory formation and storage, and in the pathogenesis of neuropsychiatric diseases and neurodevelopmental disorders [28, 71, 343]. Palmitoylation participates in 22q11.2 Deletion Syndrome- related spine structural changes and a deficit in spine density and stabilization in learning and cognitive dysfunction in that syndrome can be rescued by the ZDHHC8-dependent palmitoylation of Rho GTPase Cdc42 [77]. Loss-of-function alleles of *zdhhc9*, which encodes the palmitoyltransferase ZDHHC9 of N-Ras and H-Ras, causes X-linked intellectual disorders (XLID) exhibiting dendritic spine morphological defects [239, 344–346]. In contrast, expression of normal ZDHHC9 in hippocampal cultures is able to promote dendrite outgrowth and maintenance via palmitoylation of N-Ras and H-Ras [239]. In addition, a palmitoylation-deficient AMPAR GluA1 mutation affects spine morphology during synaptic plasticity and results in increased seizure susceptibility [70]. These studies integrate the functions of palmitoylation and regulators of spine structural plasticity, such as small GTPases, in diseases, and provide the additional insight that palmitoylation-mediated spine structural modification may serve as a valuable point of a study in understanding the pathogenesis of brain diseases and to identify pharmacological targets for treating them.

Most of the studies regarding the functional roles of palmitoylation have been carried out in brain neurons. A recent study [347] showing that Rho GTPases including Rac1, RhoA and RhoB are involved in the neurite integrity and motor neuron survival in the spinal cord in a mouse model of amyotrophic lateral sclerosis, provides hints on the role of small GTPase- regulated structural changes in the spinal cord physiology. Studies performed on spinal cord neurons have shed light on the roles of palmitoylation in spinal cord development and spinal cord-related diseases as well [90, 348–350]. Neurons in the spinal cord circuits also undergo structural and functional plasticity in response to peripheral nociceptive, tactile and proprioceptive stimuli, widely involving BDNF/NT-3 neurotrophin signaling [351–354]. In the brain BDNF/TrkB signaling promotes cytoskeletal changes and triggers structural and functional LTP [19, 48, 355], PSD-95 palmitoylation and its transport to synapses [270, 271]. Thus, it is reasonable to speculate that spine structural changes also occur in the spinal neural circuits as a component of dendritic plasticity and that palmitoylation may be involved in the processes.

## Data Availability

Not applicable.

## Abbreviations

ABHD17: α/β Hydrolase domain-containing protein 17
ABP: AMPA receptor-binding protein
AD: Alzheimer’s disease
AKAP79/150: A-kinase anchoring protein 79/150 (79 human/150 rodent)
AMPAR: α-Amino-3-hydroxy-5-methyl-4-isoxazolepropionic acid receptors
APT1: Acyl-protein thioesterase-1
BDNF: Brain-derived neurotrophic factor
Cdc42: Cell division cycle protein 42
CDKL5: Cyclin-dependent kinase-like 5
CME: Clathrin-mediated endocytosis
EPSC: Excitatory postsynaptic current
GAP-43: Growth associated protein 43
GRIP: Glutamate receptor interacting protein
HD: Huntington disease
INCL: Infantile neuronal ceroid lipofuscinosis
LIMK: LIM domain kinase
LTD: Long-term depression
LTP: Long-term potentiation
MEF2: Myocyte enhancer factor 2
NCAM: N-cell adhesion molecule
NMDAR: *N*-Methyl-d-aspartate receptor
PAK: P21-activated kinase
PAT: Palmitoyl protein transferase
PDZ: PSD-95/discs large/zona occludens-1
PICK1: Protein interacting with C kinase-1
PPT: Palmitoyl protein thioesterase
PSD: Postsynaptic density
PSD-95: Postsynaptic density protein 95
SNAP-25: 25-KDa synaptosomal protein
SynDIG1: Synapse differentiation induced gene 1
β2-AR: β2-Adrenergic receptor
WT: Wild type
